# The comparative evaluation of the effects of quercetin, α‐tocopherol, and chlorhexidine dentin pretreatments on the durability of universal adhesives

**DOI:** 10.1002/cre2.667

**Published:** 2022-10-03

**Authors:** Marzieh Moradian, Maryam Saadat, Fatemeh Sohrabniya, Mohammad Afifian

**Affiliations:** ^1^ Department of Operative Dentistry, School of Dentistry Oral and Dental Disease Research Center, Shiraz University of Medical Sciences Shiraz Iran; ^2^ Student Research Committee Shiraz University of Medical Sciences Shiraz Iran

**Keywords:** α‐tocopherol, chlorhexidine, dentin bonding agents, quercetin

## Abstract

**Objective:**

The aim of this study was to evaluate and compare the effects of chlorhexidine, quercetin, and α‐tocopherol on the shear bond strength of universal adhesives in the short (24h) and long term (6 months).

**Material and Methods:**

Ninety‐six extracted sound molars were collected and divided randomly into four groups: control (no treatment), 2% chlorhexidine, 10% α‐tocopherol, and 1% quercetin. The solutions were prepared and applied to the teeth for 60 s, followed by application of All‐Bond universal adhesive and composite build‐up. Half of the specimens in each group (*n* = 12) were tested for shear bond strength (SBS) after 24 h of storage and the other half were kept in distilled water for 6 months and then tested for shear bond strength. The shear bond strength test was performed and the failure modes were determined using a stereomicroscope. The data were analyzed using two‐way analysis of variance and Tukey's post hoc tests with *p* ˂ .05 as the significance level.

**Results:**

The results of the two‐way analysis of variance test showed that there was no significant difference in immediate SBS, and after 6 months, α‐tocopherol had the lowest SBS in comparison to the control and CHX subgroups (*p* < .05). The *t*‐test showed that the shear bond strength in the α‐tocopherol and quercetin groups was significantly decreased after 6 months.

**Conclusion:**

It can be concluded that the solutions used in this study had no adverse effect on immediate SBS. After 6 months, the CHX could preserve SBS in comparison to other groups.

## 1 INTRODUCTION

The resin–dentin bond is an important factor in the success of composite restoration. The hybrid layer is responsible for resin–dentin bonding and consists of resin tags trapped in collagen fibrils in dentinal tubules (Mehta & Subramani, 2012; Memarpour et al., 2014). Therefore, the durability of the hybrid layer depends on the stability of collagen fibrils. However, over time, enzymes such as matrix metalloproteinases (MMPs), which are present in the saliva and dentin, degrade collagen fibrils, leading to gap formation and microleakage in composite resin restorations (Delaviz et al., 2014).

Variations in MMPs have been recognized such as MMP‐2, MMP‐8, MMP9, and MMP‐20 in the carious lesions (Kiuru et al., 2021). The protein matrix of dentin is composed of 90% collagen and 10% non‐collagen proteins. The collagen proteins can be cut into fragments by MMP‐8 and further degraded by MMP‐2 and MMP‐9 demineralization of dentin by acid in the carious lesion (Moon et al., 2010).

The activation of endogenous enzymes can be done by the low pH of the self‐etching adhesive system or phosphoric acid etching or even other intrinsic enzymes. Active MMPs help to interrupt nonprotected collagen fibrils as a result of incomplete monomer penetration and play a main role in degradation of the hybrid layer over time (Carrilho et al., 2007; Pashley et al., 2004).

Etch‐and‐rinse adhesives aggressively demineralize the dentin surface up to a depth of 5 μm. Mild self‐etch (SE) adhesives partially demineralize the dentin surface only to a depth of <1 μm. Therefore, a mild SE adhesive can release a small amount of enzymes and expose less collagen susceptible for hydrolysis (De Munck et al., 2010). The current formulations of SE adhesives have integrated all ingredients into a single bottle to create one‐step systems that include the usage of a single‐step clinical application (Apolonio et al., 2017). Universal adhesives are very popular because they are easy to use and user‐friendly. They can chemically bond to various substrates such as the teeth and direct and indirect restorations (Jang et al., 2016).

A durable bond in restorative dentistry can be achieved by using MMP inhibitors, antibacterial agents, and collagen cross‐linkers (Tjäderhane et al., 2013; Zhou et al., 2019). Some materials such as CHX have these properties. According to the results of many studies, CHX increases the bond strength of the composite, especially in the long term. At the same time, there are reports that have shown the possibility of CHX's toxicity (Brackett et al., 2007; Loguercio et al., 2009; Moon et al., 2010; Münchow & Bottino, 2017). Therefore, quercetin and α‐tocopherol have been suggested as substitutes for CHX in many studies.

Quercetin is another MMP‐inhibiting substance. It is a natural flavonoid that is present in a wide variety of vegetables (Porto et al., 2018). It functions as a pleiotropic molecule with antiproliferation, antioxidant, anti‐inflammatory, and anticancer properties (I. Porto et al., 2021). Recent evidence demonstrates that quercetin can reduce the protein levels of MMP‐2 and MMP‐9 (Lu et al., 2018) and improve the longevity of the resin–dentin bond (Dávila‐Sánchez et al., 2020; Z. Liu et al., 2021; Mehmood et al., 2021).

As a natural and powerful antioxidant in the lipid phase, vitamin E (α‐tocopherol) has been proven to be a free radical scavenger. Some studies have demonstrated a positive effect of this substance on the adhesive interface durability (Daood et al., 2019, 2020; Gotti et al., 2015). Other studies have shown that antioxidants could decrease or inhibit the activity of MMPs in various tissues such as the skin or fibrosarcoma cells (Nam & Kim, 2013; Nassar et al., 2014; Pandel et al., 2013; Verma et al., 2014).

The factors affecting the time‐dependent reduction of bond strength in universal adhesives have been investigated in many studies. However, the effects of MMP inhibitors such as quercetin and α‐tocopherol on the prevention of bond strength reduction over time are still the subject of debate.

Thus, the aim of the present study was to evaluate and compare effects of dentin pretreatment with CHX, α‐tocopherol, and quercetin on the shear bond strength of dental adhesives after 24 h and 6 months. The null hypotheses tested were as follows: (1) there was no difference in the shear bond between pretreatments and (2) there was no difference in the shear bond between beginning and 6 months' time.

## 2 MATERIAL AND METHODS

### 2.1 Experimental design

Ninety‐six extracted sound molars were collected and stored in distilled water at 4°C for two months. The teeth were collected according to the guidelines of the Research Ethics Committee of Shiraz University of Medical Sciences (Protocol # IR.SUMS.DENTAL.REC.1399.049). Then, the enamel of the occlusal surface was removed using a high‐speed diamond saw under a water coolant in order to obtain a sound dentin without pulp exposure. As a result, the mid‐coronal dentin was exposed. The teeth were mounted in acrylic resin cylindrical blocks 2 cm in diameter, 2.5 cm in height, and 1 mm apical to CEJ.

To create a standard smear layer, the dentinal surface was gently polished with a 600‐grit aluminum oxide abrasive paper for 60 s under the slow flow of a water coolant. Then, the teeth were rinsed with distilled water for 10 s to remove the debris.

### 2.2 Specimen preparation

The teeth were randomly divided into four groups (*n* = 24) based on the pretreatments used: (1) Group A (control): no pretreatments; (2) Group B: 2% CHX solution (Consepsis; Ultradent, South Jordan, UT, USA) (Coelho et al., 2020); (3) Group C: a 10% α‐tocopherol solution was prepared by dissolving 10 g of α‐tocopherol gel (Sigma‐Aldrich, St. Louis, MO, USA) in 100 ml of ethyl alcohol in a standard flask (Kavitha et al., 2016); and (4) Group D: a 1% quercetin solution (Sigma‐Aldrich) was prepared by dissolving 1 g of quercetin powder directly into pure ethanol under water‐bath heating at 37°C (Li et al., 2017). The pretreatment solutions were actively applied to the dentin surface by tapping with a microbrush. After 60 s, the teeth were rinsed with water for 10 s and gently dried with a cotton roll.

### 2.3 Restorative protocol

In the bonding procedure, a universal adhesive system (All‐Bond Universal; Bisco, Schaumburg, IL, USA) was applied based on the manufacturer's guidelines in self‐etch mode. Accordingly, two coats of adhesive were used. The first coat was actively applied with a microbrush and then air‐dried for 10s. Then, without any light curing, the second coat was applied like the first one, dried, and light‐cured with an LED unit (Demi Plus, Kerr, Switzerland) for 10s at a light intensity of 1200 mW/cm^2^. Afterward, the composite resin (Aelite, All‐Purpose Body; Bisco) was built upon the dentin surface of each tooth by using a plastic mold measuring 3 mm in diameter and 2 mm in height. The excess composite resin was removed with an explorer. Then, the composite resin was light‐cured for 20 s. After removing the plastic mold, all samples were post‐cured for 20 s. The treatment agents used in this study, compositions, PH, and manufacture are shown in Table 1.

**Table 1:** Treatment agents used in this study, compositions, PH, and manufacture

Treatment agent	Composition	PH	Manufacture
Chlorhexidine	Ethyl alcohol polyethylene glycol dimethicone chlorhexidine gluconate	5.2‐6.2	Consepsis, Ultradent, USA
α‐Tocopherol	dl‐α‐Tocopherol ≥ 95.5%	‐	Sigma‐Aldrich, St. Louis, MO, USA
Quercetin	3,3′,4′,5,7‐pentahydroxyflavone ≥ 95% (HPLC)	‐	Sigma‐Aldrich, St. Louis, MO, USA
All‐Bond Universal	MDP phosphate monomer HEMA ethanol water initiators	3.1	All‐Bond Universal, Bisco, Schaumburg, USA

Then, each group was randomly divided into two subgroups for 24 h of storage and 6 months of storage (*n* = 12). The latter were stored in distilled water at 4°C for 6 months. Water was changed weekly in order to prevent bacterial growth and accelerate the degradation process.

### 2.4 Shear bond strength test

The specimens were tested in shear mode using a chisel‐shaped rod of a universal testing machine (Zwick; Roell, Z020, Germany) at a crosshead speed of 1 mm/min. The force at failure was recorded in Newtons (N), and the shear bond strength values were calculated in MPa.

The shear bond strength was the chosen method for testing the specimens in this study. Shear bond strength is a gold standard, which is why it has been widely used in previous studies (Bharti et al., 2018; Dos Santos et al., 2005; Moon et al., 2010).

### 2.5 Mode of failure analysis

To analyze the failure mode after the shear bond test, the specimens were observed under a stereomicroscope (Bestscope, Shi Jing Shan District, Beijing, China) with a magnitude of ×20. The failure modes were classified into four groups: adhesive failure (AF); cohesive failure in dentin (CD); cohesive failure in composite resin (CR); and mixed failure (MF).

### 2.6 Statistical analysis

The collected data were analyzed using SPSS software (version 25). The normality of the data was checked using the Shapiro–Wilk test. Two‐way analysis of variance was carried out to compare the interaction effect between pretreatment solutions and different times. Tukey's post‐hoc test was performed for pair‐wise comparison of the groups. A *p* value of <.05 was considered statistically significant.

## 3 RESULTS

The mean shear bond strength values and standard deviations in all groups are presented in Table 2. The data showed a normal distribution. The results of two‐way analysis of variance indicated that there was a significant interaction effect between pretreatment solutions and different times (*p* < .05).

**Table 2:** The mean shear bond strength values and the standard deviations (MPa) in all the experimental groups

	Storage period	
Pretreatment	24 h	6 months
Control	13.27 ± 2.09 Aa	11.24 ± 2.68 Aa
CHX	14.08 ± 3.70 Aa	13.11 ± 1.92 Aa
α‐tocopherol	13.02 ± 3.22 Aa	7.60 ± 4.08 Bb
Quercetin	15.77 ± 2.5 Aa	10.12 ± 3.56 Bab

*Note*: The mean values, followed by the same uppercase letter indicate no statistically significant difference in the row and the mean values, followed by the same lowercase letter indicate no statistically significant difference in the column (*p* > .05).

**Figure 1:**
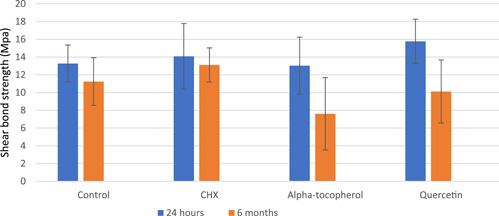
Comparison of the pretreatments over time.

There was no significant difference among the 24‐h subgroups (*p* > .05). However, a significant difference was found among the 6‐month subgroups (*p* < .05). After 24 h, the highest SBS was noted in quercetin, followed by CHX, control, and α‐tocopherol. However, there was no significant difference in immediate SBS. After 6 months, the highest SBS was noted in CHX, followed by control, quercetin, and α‐tocopherol. Quercetin and α‐tocopherol decreased SBS, and this decrease was significant in the α‐tocopherol group. Figure 1 shows the comparison of the efficacy of these materials.

**Figure 2:**
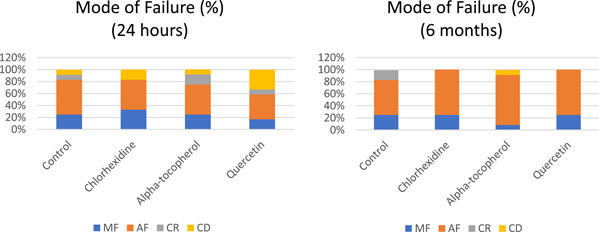
The results of the failure mode analysis. AF, adhesive fracture; CD, cohesive in dentin; CR, cohesive in composite resin; MF, mixed fracture.

Tukey's test in the 6‐month group showed a significant difference between the α‐tocopherol and control subgroups and between the α‐tocopherol and CHX subgroups (*p* < .05). Moreover, the results of the t‐test showed that the shear bond strength in the α‐tocopherol and quercetin groups was significantly decreased after 6 months. Also, the results of the failure mode analysis are shown in Figure 2. Adhesive failure occurred among all the experimental groups.

## 4 DISCUSSION

Many studies have shown that CHX increases the bond strength of the composite in the long run. However, there are concerns about its toxicity (Brackett et al., 2007; Loguercio et al., 2009; Moon et al., 2010; Münchow & Bottino, 2017). Thus, a substitute for CHX with similar effects and without toxicity should be found. Quercetin and α‐tocopherol have been suggested as substitutes for CHX in many studies. The aim of the current study was to compare them with CHX. In this study, the shear bond strength test, which is an available and standard method, was used. In addition, the shear bond strength test has been widely used in previous studies (Dos Santos et al., 2005; Josic et al., 2021; Moon et al., 2010).

In the present study, CHX with 2% concentration was chosen. In this regard, Campus et al. showed that with the use of lower concentrations of CHX, bond preservation does not occur (Campos et al., 2009).

The results of the current study showed that application of CHX can preserve the bond strength after 6 months of storage compared with the same control group. Therefore, the null hypothesis was rejected. The same result was obtained by previous studies (Bravo et al., 2017; Simões et al., 2014). The most rational explanation for these results is the ability of CHX to inhibit MMP‐2,8,9 and cysteine cathepsin enzymes, which play an important role in the degradation of the hybrid layer (Y. Liu et al., 2011).

Another material that has been discussed in recent studies is quercetin. Quercetin is recommended for improving the bond durability because of its good properties including the inhibition of ROS (reactive oxygen species) and COX‐2 (cyclooxygenase) production, leading to the inhibition of MMPs (Li et al., 2017; Lu et al., 2018; Porto et al., 2018). In addition, it is an antimicrobial agent against gram‐positive and ‐negative bacteria and viruses. These properties make quercetin a useful agent for preventing recurrent caries. It is believed that using this material in adhesive formulations leads to a desirable bonding stability, prevents secondary caries, and decreases the need for frequent restoration replacements.

In this study, quercetin improved the bond strength after 24 h compared with the other groups, but this was not significant. This is in line with some previous studies (Porto et al., 2018, 2021). Quercetin crosslinks collagen in four different forces including van der Waals, hydrogen bond, hydrophobic, and electrostatic forces (Yang et al., 2009). It might be assumed that these forces increase the bond strength immediately. However, some of them are destroyed in the long run and can no longer act as cross‐linkers. This might be the reason for the decrease in bond strength after 6 months. In the current study, the shear bond strength was decreased in the quercetin group compared with the CHX and control groups after 6 months of storage; however, this difference was not significant. This decrease can be attributed to the structural changes of quercetin that occur in acidic conditions. To explain this issue, Andrés Dávila‐Sánchez et al. claimed that dentin pretreatment with quercetin increased the microtensile bond strength significantly after 24h. This increase was not observed after thermocycling, and the microtensile bond strength decreased significantly. They stated that the decrease in pH that occurs after applying adhesives and in the process of thermocycling changed the molecular structure of quercetin and compromised its effect over time (Dávila‐Sánchez et al., 2020). This means that quercetin cannot function in an acidic environment as a collagen cross‐linker, especially in long‐term storage. This explains the results of the current study.

Kang Li and others introduced quercetin as a simple but versatile primer. They showed that quercetin/ethanol 1.0 wt% decreased the activities of MMPs in the hybrid layer. Furthermore, pretreatment with quercetin/ethanol 0.5 and 1.0 wt% killed bacteria such as *Streptococcus mutans* and inhibited biofilm growth (Li et al., 2017).

In addition, it has been stated that use of quercetin may lead to two issues. First, when mixed with adhesives, quercetin, as an antioxidant, may impair the polymerization procedure and reduce the bond strength. Second, like most antibacterial materials, quercetin is released in two stages. The first stage is the burst phase in the first few days in which a high concentration of antioxidants is released. The second stage is the tail‐release phase in the next few weeks in which antioxidant concentrations are lower than the efficacy level. Nevertheless, quercetin is hardly soluble, and it is assumed that it will not be washed away by saliva. Therefore, it might be used as a long‐term antibacterial‐releasing material. Hence, in this study, quercetin with the concentration of 1% was chosen as a dentin pretreatment to achieve optimal antibacterial performance.

Although there is a plethora of research on the antioxidant effect of α‐tocopherol on the bond strength of the bleached enamel or dentin to composite, few studies have assessed the MMP‐inhibitory effect of α‐tocopherol on the bond strength of composite to the dentin. This study showed the effect of dentin pretreatment with α‐tocopherol on the shear bond strength.

In this study, α‐tocopherol decreased the bond strength significantly in the long run. In line with this study, Gotti et al. (Valeria et al., 2015) found that adhesives doped with vitamin E may decrease the bond strength compared with the control group. They stated that hydrophobic antioxidants like vitamin E might impair the polymerization procedure and form more linear polymers. This compromises the bond strength, which could be the reason for the decreased shear bond strength in the α‐tocopherol group.

Finally, El‐Deeb et al. showed that the specimens stored in distilled water had a significantly lower bond strength than those stored in artificial saliva (El‐Deeb et al., 2016). Although the specimens in the current study were kept in distilled water, quercetin and CHX maintained bond integrity. It is assumed that these two materials could perform better in clinical situations.

The outcomes of this study cannot necessarily be generalized to other brands of universal adhesives. The results of this study cannot be generalized to clinical conditions because the specimens were not subjected to thermocycling periods and pulpal pressure. After the application of quercetin on dentin, a shift was observed in the dentin color (yellow). This observation is important for clinical considerations.

Future studies should analyze other concentrations of these materials, other brands of adhesives, and adhesives doped with other solutions. In addition, other aspects such as the degree of conversion and the polymerization rate should be evaluated.

## 5 CONCLUSION

One of the limitations of this study is that there is no difference between the control group in 24 h with use of CHX, α‐tocopherol, and quercetin. In 6 months, CHX did not impair the shear bond, showing that was no difference between the control group and the CHX group. It can be concluded that CHX could preserve SBS in comparison to other groups.

## AUTHOR CONTRIBUTIONS

Marzieh Moradian and Maryam Saadat developed the concept and theory. All authors carried out the experiment and contributed to the final version of the manuscript. Marzieh Moradian and Maryam Saadat supervised the project. Fatemeh Sohrabniya and Mohammad Afifian performed the analytic calculations and performed the numerical simulations.

## CONFLICT OF INTEREST

The authors declare no conflict of interest.

## Data Availability

The data that support the findings of this study are available on request from the corresponding author.
